# Anæsthesia—Physical and Psychical

**Published:** 1888-04

**Authors:** B. A. R. Ottolengui


### ANÆSTHESIA—PHYSICAL AND PSYCHICAL.

The Physical and Psychical Effects of Nitrous Oxide Gas; Local Anæsthesia Produced by Psychical Methods; Absolute Anæsthesia Produced in Cases of Sensitive Dentine.

BY B. A. R. OTTOLENGUI, M. D. S.

Read before the Central Dental Association of Northern New Jersey.

While I have selected as my subject Anæsthesia, it is my intention to consider the condition only as produced in one or two ways, selecting such as have special adaptability to our uses in the practice of our profession. I shall speak particularly of Nitrous Oxide Gas as what may be termed a physical agent; I will attempt to tell how to cause local freedom from pain psychically, and explain a method of obtunding sensitive dentine in carious teeth.

You all have undoubtedly learned what our text books have to tell us, and have supplemented your knowledge by practical experiences; I shall therefore give you only such opinions as I have embraced after years of practice, and recent special experiments with the gas. I have administered this agent many hundreds of times for dental operations, as well as to aid the surgeon, and I have met with many curious cases. Within the past two years I have thought to myself that whereas I have learned to act in emergencies, as we say instinctively, it would be as well to endeavor to define for myself and others the practical results of my experience. As soon as I began to cogitate on this I found myself constantly seeking for the reason why I departed from a special routine in special cases; why I administer the agent fearlessly to one person and with greatest timidity to the next. In order to find a reply, I determined to inhale the gas myself by way of experiment, and also to study effects closely in others. I shall therefore commence this paper by describing the experiments in the order in which they were made.

Exp. 1. This was with the object of determining at what stage the control of muscular effort would be effected. During inhalation I

attempted to keep up a regular and continuous motion of the hand and forearm, by raising and lowering them alternately; I also endeavored to notice other effects as they might occur. I will describe the experience in this case somewhat in detail, with the statement that it was, generally speaking, repeated in all subsequent experiments. At the first inhalation nothing was noticeable beyond the fact that the gas did not seem to be as easily breathed as the air. At the second inhalation a decidedly sweet taste, which continued to the fourth, and then ceased abruptly. Immediately at the beginning of the fifth inhalation there was a gradual feeling of warmth in the lungs, increasing almost to a sense of heat at the beginning of the sixth. Abruptly this ceased, and was followed by a slight feeling of cold, with tingling at the toes. I do not say extremities, because there was as yet no disturbance in the fingers. It will here be seen that this sensation of cold began at the sixth inhalation. I wish at this point to call attention to the fact that it required on this occasion eighteen inhalations to exhaust the receptivity of the system, and as all my experiments bear me out, I make the assertion that the exhibition of the agent may be divided into three periods, in exact proportion to the amount consumed. Thus in my description I have reached the beginning of the second period with the seventh inhalation. Now for the first time the patient begins to realize that he is about to be controlled by a power he cannot resist. (When I say "cannot," I do not mean there are no exceptions to this rule.) My experience from the sixth to the twelfth inhalation may be most aptly described as that of a naked man slowly letting himself down into cold water; though, to be more exact, the sensation of cold seems to be rising about one. In this experiment it must be remembered that the dominant idea was to keep the hand in motion, and it seemed to me that I succeeded; that is, I was at all times conscious that my hand *must* move, and that it *was moving.* Nevertheless, despite the fact that the mind was ever charged with this, it was also receiving the most exquisitely pleasant impressions. I thought that the world was slowly being left behind, and that my spirit, released from its earthly cage, was soaring up and ever up, experiencing the happiness that has so often been promised in the future state. It was at the beginning of the twelfth inhalation that I began to hear ringing sounds in the ears, which soon changed to such music as is indescribable. I was cognizant of fourteen inhalations, and after that my whole being seemed too much entranced to attend to earthly matters. I seemed to struggle with the desire to know and be able to recall my experience, and with the wish to abandon myself to the ecstasy of my new or changed being. At last all earthly ties were severed, and in fancy I was living a lifetime, when sharply and suddenly I awoke to the realization that I was again in my office, rather confused, but still enthralled with the rapture of having for a time been freed spiritually. This, as I say, was my experience then, and is always. There is one thing I must notice, however, and that is that I always awake astounded that years of time have not passed. It is also some time before I care to make any effort at either motion or speech; in fact, the tongue seems thick, or the vocal chords paralyzed for many seconds after I am restored to consciousness of surroundings. As soon as this occurred in the experiment, I questioned the gentleman who administered the gas and learned to my surprise that I had *continued the motion of the hand as long as I inhaled, and ceased abruptly as soon as I breathed one draught of atmospheric air.* I now propose giving the bare facts brought out in the experiments and make comments and deductions afterwards.

Exp. 2. An effort to test hearing. I instructed the operator to count aloud during the administration. I heard from one to fifty-three in numerical rotation, and then came forty-six, forty-seven, forty-eight, fifty-one, fifty-two, twenty-nine, thirty, one hundred and eight, one hundred and nine and one hundred and fifteen. As soon as I found that the numbers came to me irregularly, I concluded that my hearing had been modified, and dismissed further thought of the matter. Upon my awakening, however, I learned that the operator had really counted as I heard him. This I consider a fortunate accident. Had he obeyed my wishes, and I therefrom concluded that I could hear up to any point I might name on awaking, it would not be really proven, for this reason. I am satisfied that *the intellect* is never entirely controlled, if at all, and, therefore, in the endeavor to follow his counting I might receive the first impressions objectively along the auditory tract, and so getting accustomed to the intervals, continue the counting after the paresis of the auditory nerve occurred, and thus be able to approximate the point actually reached in the counting.

Exp. 3. An attempt to study the effects on sensation, sight and hearing. Result.—I heard counting up to one hundred and fifty-six, the actual count being one hundred and sixty-eight. The sight was tested by watching a view from the window, and seemed to have been undisturbed. Sensation was tested by pricking the hand. There was a gradual lessening of feeling, continuing to diminish till it was gone entirely at the eleventh inhalation, or the end of the second period.

Exp. 4. A man aged twenty-one. I counted slowly up to fifty. The patient reported that he heard forty. At forty his lips and eyelids became blue. I pinched his hand during the experiment, and he reported a cessation of feeling at thirty. The patient held the inhaler, and his arm did not drop, even after removal of the mouth piece. He became violently convulsive as soon as the inhaler was removed. He was allowed to recover gradually, and the twitchings continued until he regained consciousness.

Exp. 5. Same patient, with an interval of only five minutes. Fifty-eight was counted (faster than before). He heard up to forty. His hair was pulled during the administration, and his report was "no feeling after twenty-eight or thirty." I considered him insensible to pain at twenty-eight, the twitching of the eyelids, which had been caused by the pain, having ceased at that point. He passed urine copiously. Not to take up this again, I will here say that I have known this to occur so often that I deem it advisable that the bladder be emptied before taking gas.

Exp. 6. Not being satisfied as to the correctness of the result of the experiment as regards sight, I next took the gas myself with the special design of settling this question. I instructed my assistant to take hold of the cord of the window curtain, and as soon as he thought I appeared about to succumb, to commence pulling the curtain up and down. The result was that I made the discovery that the sense of sight departs suddenly, nothing occuring till at the twelfth inhalation. At the end of the second period I saw the gentleman move his hand up on the cord and take a firm hold as though about to begin the movement; I recollect that I then glanced up into his face, and saw his eyes turned towards the window; I therefore turned my eyes back to that point, expecting to see the curtain move, but at that instant vision ceased. I learned afterward that he commenced moving it at the fifteenth inhalation. The apparent discrepancy of results in these two experiments I will explain later.

Exp. 7. Subject, self. The operator was instructed to continue counting even after removing the inhaler, in order to test the duration of paresis of the auditory nerves. I heard the counting up to ninety-six, the next sound being one hundred and seventy-two. The inhaler was removed at one hundred and sixteen. The up and down motion of the hand was kept up until the inhaler was removed.

Exp. 8. Patient, a lady, aged twenty-two. Counted up to one hundred. She heard ninety-four. Motion of the hand ceased at seventy-eight. Counted one hundred and seventy-five before consciousness returned. At one hundred and twenty-five the motion of the hand recommenced, and kept up till she was thoroughly awake. During the interim a slight tremor was present, as though an effort was being made to continue the motion, although ineffectual. The cessation of motion corresponded with the first indication of cyanosis in the face.

Exp. 9. A case in actual practice. Female, age twenty-two. Gas was administered for the purpose of removing a pulp, Dr. F. Bradly, of Newport, being present and assisting. I directed the patient to beat with the hand as long as possible. She did so up to the last two inhalations, and then followed a trembling of the member, as in the last case. I opened into the pulp-chamber with the engine bur, and then with a broach removed the pulp. At this point her eyes opened and signs of returning consciousness appeared. I changed the bur, replacing it with canal reamer and reamed out the canal. Upon the return of consciousness the patient reported no pain.

Exp. 10. Same patient. Removal of a tooth; twenty-seven inhalations, the motion of the hand as requested being kept up to the twenty-fifth. After extraction the patient was allowed to remain undisturbed, and timed by a watch, two minutes being allowed to elapse. During this time the patient talked aloud and seemed conversing with some friend. This case is specially interesting. In all my experience I have had but two patients who talked, the first being one who feared she would "tell her secrets," and in fact revealing the name of her lover while under the influence of the gas. In the present instance I had related this fact to the patient just prior to administering the gas, and also told her to think of some one of whom she was fond. The result was the repetition of a conversation had with her intended the night previous. She told me afterwards that she thought she was at home, and going over this scene, and when I related what she had said, she claimed that it was almost verbatim what had passed. It can scarcely be imagined how singularly this sounded. A girl, apparently asleep, and yet speaking aloud with invervals between, as though another was conversing with her.

These are not all the experiments I have made, but they will suffice to bring into prominence the points I wish to make. What then do we learn, and what useful deductions can we draw from these facts? Bear in mind, first, that in all the universe we find no two creations, animate or inanimate, identical, and that, therefore, it is manifestly impossible to state dogmatically that a special agent will accomplish a special result. I have found patients particularly susceptible to this gas, and others on whom no amount taken into the lungs would produce the least anæsthetic effect. Nevertheless, for general purposes, the statements I make in regard to its action will be found true, the limitation being only in degree in correspondence with the individuality or soul power of the subject. I have investigated the action of the gas on special sense only as to sight, hearing and touch, taste and smell being matters of little moment to us whilst operating, though it would of course be interesting to extend research in this direction. I have also tried to learn the effect on the will and on volition, when I introduced a motion of the hand during inhalation. The result is that impressions along the nerves of sight and hearing cease abruptly, whereas the sense of touch or feeling is overcome by degrees. Again, sight and hearing are only lost in the third stage, whereas feeling begins to lessen at the beginning of the second. These are points of great usefulness to the operator, and if thoroughly understood will enable him to administer the agent with satisfactory results to almost all classes and conditions of patients.

How then shall he be able to determine the boundary lines of the different stages? Not by counting inspirations, for one patient will take only ten, where another will take as many as thirty-five breaths before stertor commences. The chief object in view is anæsthesia—in its strict derivative sense, freedom from pain—and this has been demonstrated to occur at the end of the second stage. Operations can be performed with perfect safety at this time, and with certain immunity from pain. How shall this moment be recognized ? In a very simple way. Having divided the effects into three periods, as the result of my personal experiences, I next endeavored to find the outward signs which would be coincident with the end of the second period. This I accomplished by watching the faces of subjects, counting aloud, and causing continuous sensation by pulling the hair, pricking, etc., and then by asking at what period of my counting the cessation of feeling had occurred. The sum of these inquiries is, that the patient ceases to feel at the exact moment when the first appearance of blueness occurs, and this will generally be first detected in the capillaries of the eyelid. If for any cause the operator has any anxiety as to the safety of his patient, let him press the agent no further, but remove the inhaler and operate swiftly. It is indeed a safe rule never to go beyond this point unless there be special reason for desiring a prolonged sleep, as, for example, in removing a number of difficult roots. I have further shown by the examples cited that as the sense of touch is the first to be overcome, it is reversely the last to be restored, and therefore it follows that the operator may continue even after the eyes begin to open and the patient to show signs of consciousness.

I will here insert a case from actual practice, showing the truth of my statement in regard to the time at which the sensibilities are benumbed. Patient, a female, aged twenty-five. Operation to be performed—the stretching of the sphincter of the rectum. I have frequently given the gas for the gentleman who operated in this instance, and on former occasions had proceeded as follows: I would allow the patient to take all the gas possible, and then giving the sign to operate, turn the gas off and let air be breathed until the blue discoloration in the face had almost passed away, and then give the gas again, thus alternating air and gas as long as required by the surgeon. In this way I have kept a patient under influence for forty-five minutes, rectal tumors being removed, three in number. In the present instance I gave the signal to operate as soon as the first blue color appeared around the eyelids, and by the time the patient had become fully cyanosed the work was accomplished. She reported absolutely no pain or consciousness of the operation. I will state, too, that it has been my observation that the pain consequent on stretching this sphincter is harder to overcome with gas than the actual use of the knife.

As to hearing, there is little to say beyond the fact that it should be remembered that the patient can hear almost as long as he continues to inhale the agent, and therefore it is possible to give continued directions as to manner of breathing, etc. In the last case, after giving the signal to the surgeon and he had commenced to operate, my patient opened her eyes. I at once directed her to close them, and she obeyed promptly, proving not only that she heard, but that the power to obey still lingered. This knowledge of the continuousness of hearing can be made very useful in soothing disturbed emotional conditions.

As to sight, it will be remembered that in one case I reported that it had not been affected, and in the next that it had been entirely obliterated. In the first instance I gazed on a still view, seen through the window. Thus, although the sight was lost, it went so instantaneously that its going was not a cognizable fact. Its return was similarly quick, and as the view was the same there could be no knowledge of the interim. This, however, proves an interesting fact. In alcoholic anæsthesia, when the patient is recovering from his stupor, he "sees double," to use a common phrase. Why is this? The explanation is as follows: Man has two eyes, and each is capable alone of conveying impressions to the brain, the result of which we call sight. Nevertheless, these two eyes see but one object. This is accomplished by focusing. There is accommodation of the lens in each, which is nicely adjusted to meet required distance, and besides, the eyeballs are so turned by the recti muscles that the two lines drawn from a given point on the object to the lenses will be of equal length. The drunken man fails in this because there is a paresis of the recti muscles. If this were true in the case of the gas, I should readily have discovered a difference of vision in the first instance.

Before coming to the consideration of psychical effects, there are one or two practical points of which I wish to speak. It should be always remembered that if the gas is administered from a reservoir, as is the best method, the first breaths will be difficult, since some force must be applied in order to make it come through and along the tube. Therefore, this should be explained to patients who are apprehensive of danger, otherwise, as soon as it is perceived that there is a difficulty in breathing, the idea of being suffocated will be entertained, and strenuous efforts sometimes made to remove the inhaler; if successful in this it may be difficult or impossible to induce a second attempt, and if the inhaler is forcibly kept in place, your patient may cry, groan, or even cease breathing, the breath being sometimes held almost to the point of asphyxiation.

Another important matter is the prop or gag. Not very long since a prominent operator in New York was sued by a woman who claimed that she had had a clicking jaw ever since she took this agent at the office of the defendant. She was more or less ridiculed in court and by the press, and lost her suit. I do not mean to pass judgment in this case, for I am not in possession of details and facts, and besides I know the gentleman to be a skilled and experienced operator. But I think there is a profitable lesson to be learned here. Why do we use the prop at all? Because we do not press the agent to the point of relaxation, nor is it necessary, as it is claimed can be done by Paul Bert's method, and therefore we extract whilst the jaw is rigid. But there is more than simple fixity of the jaw, for in that case we still would not need the prop, for the mouth would remain open. There is a violent contraction of the masseter muscles. Now think a moment of the exact position of these wide and powerful muscles, and does it not become apparent that there may be some danger in inserting a prop between the jaws? The farther back it is placed the longer becomes the lever to pull the condyles out of position. Place the prop between the front teeth, and it would be plainly impossible to affect this joint and the ligaments about it. It is not always possible to do this exactly, as the prop would most likely be in the way, but care should be taken not to place a prop back of the centre of the fibers of the masseters. The bicuspid region is perhaps safe enough. A further precaution can be taken in making the props with cushioned ends, using hard and soft rubber.

In the article on the agent in the "American System of Dentistry" I find one remark to which I wish here to reply. "The pulse and respiration are sometimes accelerated at the beginning of the inhalation, and it is not yet possible to determine to what extent these phenomena are due to the action of the gas." They cannot be attributed to the gas at all, except subjectively. We have here the simple result of anxiety, which is a mild form of fear. As to the pulse, it is an admitted fact that fear produces acceleration of the heart's action. Dr. Hack Tuke says: "Acceleration of the heart's action through the sympathetic nerves is the most frequent and obvious result of emotional excitement." Later, I will givė a good example of this from actual practice. As to the disturbed breathing, that is manifest from the very manner in which we see it occur. The anxious patient inspires fitfully, for fear of taking the agent, then holds the breath as long as possible, expiring only enough to allow a new breath which shall receive only a little of the terrible vapor. Thus these short breaths seem accelerations. Mark the difference. A moment later, when the agent begins to act, confidence is restored by the pleasurable sensations produced, and respiration becomes strong, and if then, as we here see, the emotion plays such an important part in anæsthesia, how necessary it is for us to consider and study the principles which surround the question, in order that we may as often as possible send our patient into the happiness of pleasant dreams, rather than the mirage of distorted emotions occassioned by dread! Here comes in the importance of keeping the eyes shut, possessed as we are with the knowledge that sight continues so long; and of course so long as it does endure it is causing impressions, more or less disturbing the peace of the spirit. Secondly, having used our utmost endeavors to calm our patient by our assurance of safety and a pleasing experience, how valuable becomes the knowledge for psychic treatment that he can hear, for can we not thus still further endeavor to dispel any lingering doubts by quietly remarking to our assistant (not to the patient) that he is a "remarkably good subject," that "he takes the gas excellently," etc !

There is now another aspect to be considered. Is it not possible that the condition we call anæsthesia may be induced by purely psychical agents? The answer is, undoubtedly, yes! And there are two classes of this phenomena. First, those caused without intent, and second, where an operator directs such psychic agents with the direct intention of producing this condition. Instances of the first class are seen in hysteria and catalepsy. Very remarkable cases are cited by Dr. Tuke in his work, "The Influence of the Mind on the Body," which I recommend as most profitable reading to all who would pursue this subject. Perhaps the most complete psychical anæsthesia is that caused by religious fanaticism, as exhibited in the convulsionaires at St. Medard. One of these, Nisette by name, was

"struck on the head with a log, and then had the four extremities pulled in different directions. Two men stood on her body, then two on her back, whilst others dragged her up by the arms and gave her the strapado," etc., inflicting every kind of cruelty possible in the attempt to force her to admit that she could feel, but unsuccessfully.

It is plainly with the second class, however, that we are most interested. Psychic anæsthesia had begun to take a prominent place in surgery, when chloroform was discovered to produce the desired end with such readiness that surgeons at once dismissed all investigations of the condition as superinduced by those methods, which may be all classed under the title, mesmerism. That these phenomena were real, is established abundantly by the fact that a hospital was founded in Calcutta and flourished for ten or twelve years, during which period Dr. Esdaille, the eminent surgeon in charge, operated with brilliant success, painlessly performing some of the most heroic operations known to surgery. Such practice, however, would not be most convenient for practical use by the dentist, although Dr. Esten, of Providence, used this method as early as 1837, in extracting. Then, too, in very susceptible persons, perhaps rare cases, anæsthesia may be induced simply by pretending to administer a known agent. Dr. Tuke tells of a case of this kind, but I must abbreviate it instead of quoting verbatim. A young woman applied to a London hospital, complaining of three sebaceous tumors of the scalp. It was decided to operate, and she was taken to the proper room and placed in position. At the last moment it was discovered that the chloroform bottle was empty, and while an assistant was sent to replenish it, the operator determined to accustom his patient to the mouth-piece, and placing it to her mouth directed her how to breathe. She did so, of course not knowing that there was no agent present, and after a few inhalations passed into the usual condition. The operator noticing this directed the surgeon to proceed, and one tumor was then removed. Wishing to make a test, he then said: "Wait a moment; she awakens." At once she showed signs of recovering, but by simply replacing the mouth-piece she returned to the unconscious condition, and so remained whilst the operation was completed and the dressings applied. I have myself experimented in this direction, but not often enough as yet to report with certainty. I have, however, succeeded in removing a tooth with little pain, and have generally found my patient in a dazed condition, though not unconscious. But I have not pressed these experiments, because if the subject agrees to take the gas I do not hesitate to give it. Thus it is only where I find a patient who so fears the gas that he will not consent to take it that I really need a psychical method of performing the operation, and this I can do very readily, as I shall now describe. And it is at once seen that in such a case I could not cause the condition by pretending to give the gas, because that requires the consent on the part of the patient to have the inhaler placed in position.

Before explaining this method, I must describe one case in which total anæsthesia was induced without intending it. The subject was a highly nervous, ignorant woman. Placed in the chair and the inhaler applied, on the second inspiration she became rigid, ceased to breathe and was plainly unconscious. The extraction was swiftly accomplished, and it was twenty seconds by a watch before she stirred, when she declared that "the gas was loike goin' to hiven intoirly." There is, of course, a principle involved in such a case as this, and it is contained in the words of John Hunter, when he said, "I am confident that I can fix my attention to any part until I have a sensation in that part." Thus the effect of attention. If to this is added expectation of a certain result, the effect will be modified in that direction. Dr. Tuke, in his work already alluded to, explains most thoroughly all the scientific facts behind this statement, and testifies to its verity by very many examples. I have not the time in so short a paper to expound these truths, but having accepted them myself, I proceeded to put them into practice, and perhaps the result of my experience will, after all, be of more practical interest, especially as each can study the principles for himself.

My method is simplicity itself, so far as act is concerned. I saturate a piece of cotton with a little colored water and apply it about the tooth, with the result that in two or three minutes' time I am enabled to remove the tooth or root without inconvenience to my patient. It would scarcely do for me to stop with this bare assertion, for should you follow this prescription it is more than likely that there would be a total failure. Why ? I have said that the anæsthesia, though only local, is psychical in character. Colored water and cotton would scarcely constitute a psychical remedy. Perhaps the best way to explain the working of my method is by an illustration from practice. Some months ago a lady made an appointment to have ten roots in the lower jaw removed, and agreed to take gas. The appointed hour was ten o'clock. On reaching my office at nine I found her pacing the floor. "In pain, madam?" asked I. "Oh, no! But, doctor, I have not slept all night, thinking about that gas. I am afraid if I take it I shall never wake up." "Under those circumstances I would not advise you to take it." "But what shall I do? I could never have them out in any other way." "Oh, yes," I replied, "I know just the way out of your trouble. I have a very powerful remedy, one or two drops of which if applied to the neck of a tooth will so deaden the parts that it can be removed without pain." "What nonsense, doctor; you are fooling me." "Not at all," this said with perfect gravity, "it is a fact, just as I have related." "But what can it be that can act so wonderfully?" "It is called Goutte d'or." "What does that mean?" "It means Golden drops. This is a preparation I get from Paris. Shall I show it to you?" I then produced my bottle, which is a curiously shaped salts bottle, green in color and having a silver top covering a tiny glass stopper. This, of course, is intended to have an effect on the imagination. In this case my patient, after looking at and smelling my Golden drops, agreed to come again at the appointed hour, and she did so, allowing me to remove the ten roots by simply placing the preparation on each one alternately. I always do this, though in her case the special reason was that she asked me if, like rhigolene, it might not leave a sore gum afterwards. I at once assured her that the effect was so fleeting in character that it would be necessary to operate rapidly after removing the application, and then renew it for each tooth. Having always in similar manner applied my psychic agent, really existing in my manner and conversation, the actual application itself is useful in that it directs attention to the point to be operated on. Then having taken my watch off my chain and laid it on my stand, explaining to the patient the necessity of exactness in timing the duration of the application, I place a few drops on the cotton and apply it, observing strict silence, and persistently allowing three minutes to elapse before extracting. I am happy to be able to say that thus far, out of half a hundred cases, I have not had a failure. I wish to exhibit the cast of one, to show that I do not select easy cases. This patient, a lady, aged forty-five, applied to me to remove the two last teeth in the upper jaw, as they were excessively loose. She refused to take gas, and I readily removed the two teeth, one the second molar and the other a supernumerary, though at first I took it for an abortive wisdom tooth. After the extraction she informed me that all were not out. Imagine my surprise, on exploring, to find the cusp of another tooth. I endeavored to extract this, and worked arduously for several minutes, using different forceps and utterly failing to even loosen it. At length she refused to endure more. I tried to persuade her, but in vain. Like a flash of inspiration, I seemed to hear the magic words "Goutte d'or." At once I explained its virtues to her, at length gaining her confidence in its power, and applied it for the space of four minutes, telling her it was a minute longer than usual. I then went boldly up with a bayonet molar forcep, obtained a good grip and removed the tooth without pain, as she reported.

One more case, because of the effect produced on circulation, which I promised earlier in this paper to describe, and which I accidentally discovered. I had the very bad root of a first bicuspid to remove. Having applied the Goutte d'or, I was holding the same against the parts with my forefinger, when I noticed that I could feel the pulsations of the superior coronary artery. This was a good opportunity to test her pulse without her knowledge. I therefore counted, with the following result: First minute, ninety-five, irregular. Second minute, ninety-eight, irregular and slightly faint. Third minute, one hundred, quite irregular, and at times very faint. The root being removed without pain, proving to be bifurcated, I left her for five minutes. On my return I asked to look at the parts a moment, and took the chance to time the artery again, without telling her of my intention so to do. The result was, for one minute eighty-two pulsations, and all firm and regular.

In another case, that of a miss of fifteen years, the pulsations were one hundred and fifteen, one hundred and seventeen, one hundred and seventeen, for the three minutes, while the normal pulsations were subsequently found to be ninety-eight.

As an illustration of the fact that this principle as applied to dentistry is not new, I desire to quote from an ancient work which was recently shown me, written in the Latin tongue. The title is "*De Superstitione et Vinculis Demonum,*" which means, The Superstitions and Tricks of the Demons. The body of the work is devoted to a description of some of the practices of the ancient Magians. In turning over the pages I was attracted by a short paragraph with the heading, "*Dentium dolore,*"—Tooth-ache. Then follows: "*Maecubales Hebrarum digito tangunt dentium ternis diebus mane, ter verba hoc proferentes " Deus Abraham, Deus Isahac, Deus exercituum, liberet te a dolore dentium.*"

Freely translated this means: "The Hebrew priests touch the tooth every three days, early in the morning, repeating these words three times: May the God of Abraham, the God of Isaac and the Lord of Hosts deliver you from tooth-ache." A very good example of what is now known as Christian Science, or cure through prayer, and in practice, too, before the advent of the Saviour.

The next paragraph is even more interesting: "*Germanos apud hoc patet experimentum; patiens album filum in acum positum agenti porrigit, aut per alium praeceptori transmittit, petitque si superiori dentes, aut inferori torquentur, clamque notatis in schedula hisce quinque verbis N. A. W. G. E. Si dolore inferiori parti singulas primae literae partis in extremitatem pungit filum ex opposita parte extrahens, iterum atque iterum, dolores cessatione repetens.*" This translated, in substance, is: "Among the Germans we find this experiment. The patient presents the operator with a piece of white thread in a needle, or sends it by some other person, and he, the operator, asks whether it is an upper or a lower tooth which troubles, and having plainly written on a piece of paper these words, N. A. W. G. E. if the pain is in the lower part he pierces each part of the first letter with the needle, pulling it through violently from the opposite side, repeating this again and again until the pain ceases." I regret very much that I cannot translate the magic words which are only given in initials, for it might enable us to try this simple experiment. However, that it must have been efficacious we cannot doubt, for be it observed we are told the operator repeated his trick again and again till the pain ceased. Jesting aside, it must be admitted that tooth-ache was often, if not always, treated by a "charming method" among the ancients. Dr. Tuke quotes from Dr. Ranieri Gerbi, a professor at Pisa, who relates the wonderful therapeutical effects possessed by an insect which he calls "*Curculio anti-odontalgicus.*" It was only necessary to squeeze this insect between the fingers and apply to the tooth to bring relief. Dr. Gerbi relates that, by this process, which clearly owed its success to the imagination, he cured four hundred and one cases out of six hundred and twenty-nine.

A similar practice is still in existence in the Southern States, especially among the negroes in the Carolinas. I cannot tell if the insect is the same, but there they use a small bug commonly found on rose bushes and other flowering plants, and widely known as the "Lady-bug" or "Lady-bird," of which children often sing a little verse. This bug, if squeezed between the fingers, and then rubbed on the gums, is considered a specific.

Although I fear that I have already too long tried your patience, I still shall not close without describing to you how I cause a local anæsthesia in cases of sensitive dentine. In endeavoring to accomplish this I began by theorizing, something as follows :—

The investigations of recent histologists in the laboratory of Dr. Heitzmann, have demonstrated that not only in the dentine, but in the enamel as well, there exists a fiber of living tissue. Moreover, that even the basis substance of the dentine is everywhere traversed by a minute reticulum of similar tissue, which commences as spines from the fibers, and which interlace and anastomose in every direction. Further, that these fibrillæ, if not nerve, are neural tissue, for it can be plainly seen that in appearance at least they are identical with nerve. The dentinal fiber is continuous with, or in juxtaposition to, the odontoblasts of the pulp. Undoubtedly, then, this is the channel through which the sensations resulting in that disturbance of the nerve centres which we call pain is transmitted when we cut across these fibers with our sharp instruments. It has been found almost impossible to antagonize, or more correctly speaking to control, this sensitiveness with any agent yet known. Partial effects have been produced, but with no one method within my knowledge has perfect immunity been obtainable, allowing uninterrupted operation. The methods giving the best results are those which embody evaporation of the moisture in the tooth. This is easily seen. The hot air syringe has its advocates, and chloride of zinc is perhaps the most reliable topical remedy. What have two such dissimilar agents in common? The hot air evaporates, and the zinc crystals accomplish the same result by virtue of affinity for water. The explanation of this leads to the end. The fibrillæ do not fill the tubuli. This is manifest to the eye of the microscopist, and if further evidence were needed it lies in the fact that the fiber is in appearance like a piece of string with beads put on it at short distances; thus, even if the beaded parts touch the walls of the canaliculi, the parts between cannot. The tube not being filled by the fibers, contains water, which enters by capillary attraction. This is probably taken up mainly from the fluids of the mouth, for when we isolate teeth with the rubber-dam, even those which are not carious soon show by a changed appearance that evaporation has occurred. This also tends to prove that such evaporation is possible. What useful end can this serve? If it is the fibrillæ which transmit the sensation of pain to the dental pulp, if they can be so contracted or benumbed that we, in the first instance, do not cut them, or in the second, find them incapable of normal action, we have practically overcome our enemy.

This may be done as follows: Apply the rubber-dam, dry the cavity with bibulous paper, and then insert a pledget of cotton saturated with absolute alcohol, selected on account of its affinity for water; next direct a series of blasts of hot air to the cavity, persisting till the parts become whitened, or thoroughly dry, usually taking as long as three minutes. It may be as well to mention that what is usually sold as a hot air syringe is not the best to use, from the temptation to make the application of the blast continuous, which will result in pain. It should be applied intermittingly; remember that evaporation is what we are seeking, and not the heating up of the tooth, and this may be accomplished as well by stages as by continuous heat, since the supply of moisture is cut off by the dam. When satisfied that the water is evaporated, throw on a continuous spray of ether. This will at first, in about forty per cent. of cases, cause pain, which will, however, almost at once begin to lessen as the spray is continued, and in the end it will be found that all sensation will be controlled. The tooth may now be cut almost at will, and with no change in sensitiveness in consequence of entering the dentine, even to a considerable depth, for undercuts, anchorages, etc.

I have taken up so much of your time that I shall not endeavor to theorize on this to any extent, further than to say that whilst I do not think the contraction of the fiber would entirely account for the lack of sensation (for we cannot believe that it could be made to contract one-half its entire length, which it must do to allow a deep drill hole without feeling, if this were the only explanation). I believe that this contraction has much to do with the result. However, the result is what will most interest our patients, and this I have found to be uniform. I have used this method now in nearly fifty cases, and have not had a single failure. One case from practice will demonstrate how the method succeeds. The patient presented a tooth which was so sensitive that on drying out with a piece of bibulous paper she fainted. At her next visit I explained what I wished to do, and was able to thoroughly prepare the cavity, and then fill it with gold, without the least pain. This method is also most happy in its results in cases where from recession, the necks of the teeth, especially of cuspids, have become sensitive. In such cases, where it is impracticable to apply the dam, cover the gum tissue with a thin solution of pink gutta-percha in chloroform. This, by evaporation, will leave a film of gutta-percha which will effectually keep the parts dry. I may say interjectively, that fillings may be inserted in this way at times, with much comfort to both operator and patient, thus avoiding the painful clamp. After this film is hard, use the alcohol, hot air and ether spray as directed, and then burnish the surface with a rapidly revolving corrugated burnisher in the engine. If there is softened dentine present, cut away with a gold finishing bur, thus avoiding the danger of going too deep, and follow with the burnisher. I may say that in this way I have successfully operated on the most excruciatingly sensitive teeth, and this state of hyperæsthesia has not supervened.

In conclusion, I can only say that chimerical as some of these statements may appear, they are based on close observation, though it may be that I have improperly understood some of the conditions which it has been allowed me to see. Nevertheless, dim as my light may be, it has illuminated my professional path to an extent for which I am profoundly grateful, and I thank the Almighty Creator that even these dim rays have been allowed to reach and warm my soul, and thus put that inspiration into my endeavor, without which no man may hope to progress.

Thanking you, gentlemen, for your courteous attention, I beg leave to submit my paper, with the hope that if there be any present who can point out to me my errors, he will do so to my benefit.

